# Workplace Phobic Anxiety as a Mental Health Phenomenon in the Job Demands-Resources Model

**DOI:** 10.1155/2017/3285092

**Published:** 2017-11-29

**Authors:** Michela Vignoli, Beate Muschalla, Marco Giovanni Mariani

**Affiliations:** ^1^Department of Education Studies, University of Bologna, Via Filippo Re 6, 40100 Bologna, Italy; ^2^Applied Psychology, SRH University of Applied Health Sciences, Neue Straße 28-30, 07548 Gera, Germany; ^3^Department of Psychology, University of Bologna, Viale Berti Pichat 5, 40100 Bologna, Italy

## Abstract

**Purpose:**

Anxiety-related problems at work are a serious problem in the occupational context, as they come along with sick leave and problems in work participation. The aim of this study is to analyse workplace phobic anxiety in nonclinical context using the Job Demands-Resources model.

**Methods:**

The study involved a sample of 739 workers from a retail company, mostly with permanent contracts. Structural equation modelling analyses were performed using AMOS software.

**Results:**

Both the health impairment and motivational variables in the JD-R model were significantly related to workplace phobic anxiety and subsequently to absenteeism, specifically, exhaustion mediated between perceived job demands and workplace phobic anxiety and work engagement mediated between perceived job resources and workplace phobic anxiety. Moreover, workplace phobic anxiety was significantly positively related to absenteeism.

**Conclusions:**

Results suggest that workplace phobic anxiety is a specific concept and an important issue in organizations for both workers' health and the organizational costs linked to absenteeism. Supervisors and occupational physicians should be aware of workplace phobic anxiety, especially when workers are on sick leave often or for long periods.

## 1. Introduction

Nowadays new, broader and stronger sources of work-related stress can increase an individual's vulnerability to more serious mental health. Anxiety symptoms are serious and critical problems in the occupational context and they can be associated with stress.

Anxiety and depressive disorders have been found to be among the most commonly diagnosed mental disorders, affecting millions of people in many of their daily aspects of life [1]. About one-third of the general population suffers from mental disorders [2].

The last survey conducted by the Anxiety Disorders Association of America [3], highlighted how stress and anxiety can be related to workplace. The findings of this survey showed that anxiety at work could influence workplace performance, relationships with colleagues, quality of work, and relationships with supervisors. Furthermore, the workplace could affect anxiety through pressure over deadlines, interpersonal relationships, and dealing with issues or problems that could arise during the performance of work activities. Concerning the relationship between anxiety and work, the concepts of “workplace-related anxieties” and “workplace phobia” appear as new work-clinical concepts [4]. Particularly, workplace phobia is the most severe form of workplace-related anxiety; it can affect an organization's performance since it is related to absenteeism. In order to deepen the knowledge of this clinical concept, which to now has never been studied in terms of the potential work environment aspects, the main aim of this study is to analyze workplace phobic anxiety in the context of the most used model concerning psychosocial risk factors and stress at work: the Job Demands-Resources model [5].

### 1.1. Workplace Phobic Anxiety or Workplace Phobia

Workplace phobia has been defined as “characterized by a classical phobic anxiety reaction concerning the stimulus workplace. It occurs in a panic-like reaction with physiological arousal when thinking of the workplace or approaching. The person shows clear avoidance behaviour towards the workplace. Due to the symptoms, there must be severe subjective suffering and/or impairment carrying out daily duties at work” ([4], p. 46). Furthermore, as reported by Muschalla and Linden [6], referring to the Diagnostic and Statistical Manual for Mental Disorders V [7] and the International Classification of Diseases and Related Health Problems [8], workplace phobia can be categorized among the anxiety disorders, particularly as a specific phobia (DSM: 300.29; ICD-10: F40.298). Using a differential diagnostic approach, it has been demonstrated that workplace-related anxieties can be distinguished from conventional anxiety disorders [9]. Haines and colleagues [10] published the first experimental study that proved the existence of workplace phobia. The aim of their study was to determine whether a group of people who exhibited avoidance in the workplace could be identified in terms of their psychological and physiological responses to stressful work events. The criteria for workplace phobia diagnosis in the study were (a) self-reported intensive fear when approaching or passing the workplace; (b) inability to enter the workplace because of severe anxiety symptoms; (c) reduction of physiological responses when leaving the workplace. Results showed that all participants reported an increment of psychophysiological arousal and psychological response to stressful work events in comparison with neutral events. But the work-phobic group reported higher heart rate response and subjective reports of fear that distinguished them from the other groups.

Besides this, other studies (e.g., [9, 11]) found that workplace-related anxiety and workplace phobia are different to conventional anxiety disorders [4] and can be distinguished empirically. Within groups of persons with clinically relevant anxiety, there are (a) persons who have non-work-related anxiety disorders, (b) persons who have non-work-related anxiety disorders and workplace phobia, and (c) persons who only suffer from workplace phobia (without any other non-work-related anxiety disorder).

Workplace phobia must be distinguished from other concepts usually studied in the organizational psychology field, such as mobbing (bullying) or burnout.* Mobbing *characterizes negative behaviours carried out against an individual employee frequently and over a prolonged period of time by colleagues or supervisor [12]. Mobbing is thus a job condition but not an illness.* Burnout* is a phenomenon characterized by emotional exhaustion, cynicism, and reduced professional efficacy [13]. None of these conditions are similar to workplace phobia as they are not illnesses, and they are not specific in symptomatology. Workplace phobia is a phobic anxiety syndrome characterized by physiological arousal when confronted with the stimulus workplace in vivo or sensu and a tendency towards workplace avoidance [4]. Due to its specific leading symptoms (work-related panic and avoidance) and its specific impairment in work ability or even leading to sick leave, workplace phobia can be described as an illness [8, 14].

In the last decade, antecedents and consequences of workplace phobia have been defined. Concerning antecedents, Muschalla [4] provided a model concerning the aetiology of workplace phobia. This model posited that four factors (conventional mental disorder; workplace-related releases; non-workplace-related events; and psychosocial stressors and personality, as well as individual mental and physiologic disposition) could lead to the development of work-related anxieties and eventually workplace phobia. Concerning psychosocial stressors, Muschalla and Linden [14] reported that workplaces could contain factors which can provoke anxiety, for example, demands for achievements, which may provoke generalized or existential worrying. For example, a perception of high workload could be one aspect related to the degree of perceived workplace-related anxiety [11].

The most characterizing consequence of workplace phobia is avoidance of the workplace and even associated public places, as the fear is to be confronted with workplace-associated stimuli (e.g., colleagues or supervisors), but also avoidance of objects or places which remind the person of the workplace [4]. Another consequence of workplace phobia, based on the fact that avoidance is the most important criterion of phobic anxiety disorders [10], is absenteeism, especially long-term sick leave [14]. The specific relationship between workplace phobia and absenteeism has been proofed empirically by two studies. One study by Muschalla [11] found that the longer the sick leave, the higher the probability of suffering from workplace phobia. Another study [6] demonstrated that individuals with workplace phobia had longer durations of sick leave compared to patients without workplace phobia.

In this present investigation, we use the term “workplace phobic anxiety,” because we did not diagnose “workplace phobia” as an illness. Diagnosing workplace phobia as an illness can only be done by face-to-face medical examination. In this study we used self-ratings only. Self-ratings do not allow any diagnosis but can describe the degree of workplace phobic symptom load.

### 1.2. Absenteeism

Absenteeism can be a coping mechanism to deal with stressful job demands, instead of merely a behavioural reaction to dissatisfaction [15, 16]. Taking sick leave could represent a worker's strategy to save energy, provide an opportunity for recuperation, and detach oneself from a stressful, nonrewarding, nonsupporting, and conflict-ridden work environment [17]. Usually, absenteeism is categorized in two main typologies: voluntary and involuntary. Bakker and colleagues [18] defined voluntary absenteeism as a function of employees' motivation. In contrast, involuntary absenteeism is defined as the inability (rather than unwillingness) to go to work, as a result of illness or other exceptional circumstances. The first one is measured by the number of times an individual has been absent during a specific period, irrespective of the length of each of those absences, while involuntary absenteeism is measured by the total length of time an individual has been absent over a specified period, regardless of the number of absence spells [18]. Despite this distinction, recent studies [19] demonstrated that the two absenteeism measures (duration and frequency) showed similar reliabilities and their association with each other approximates unity. Thus, the voluntary distinction seems to be unsupported. Therefore, based on their results, Johns and Al Hajj [19] suggested that researchers measure absenteeism in terms of both frequency and duration without making attributions about their relative voluntary attributes.

### 1.3. Workplace Phobic Anxiety in the Job Demands-Resources Model

To the present day, there have been few studies of workplace phobic anxiety in organizational settings. One study investigated self-reported work-related anxiety in a working population and found that about 5% of mentally healthy employees reported workplace-related avoidance [20]. Despite its important consequences for work productivity in organizations, workplace phobia has never been considered as a health aspect in organizational psychology models, for example, the Job Demands-Resources model (JD-R, [5, 21, 22]). The JD-R model is one of the most used models for analyzing well-being in organizations. There are two main propositions in this model. The first one is that all job characteristics can be divided into two main categories: job demands and job resources. Job demands are defined as “those physical, social, or organizational aspects of the job that require sustained physical or mental effort and are therefore associated with certain physiological and psychological costs” [5]. In contrast, job resources have been defined as “those physical, social, or organizational aspects of the job that may do any of the following: (a) be functional in achieving work goals; (b) reduce job demands and the associated psychological costs; (c) stimulate personal growth and development” ([5], p. 501).

The second assumption of the JD-R model is that it is composed of two main processes, namely, the health impairment process and the motivational process. The health impairment process assumes that long-term excessive job demands from which employees are not able to effectively recover could lead to sustained activation and overtaxing, and this may result in exhaustion, which is the central component of burnout [23]. The motivational process posits that job resources have motivational potential and could lead to work engagement [21]. In their critical review of the JD-R model, Schaufeli and Taris [23] discussed an issue referring to the distinction between the health impairment and the motivational processes. The authors claimed that despite the two independent health impairment and motivational processes in the JD-R model, it is quite imaginable that they represent two sides of the same coin. This means that when health and well-being deteriorate, motivation decreases and vice-versa. Schaufeli and Taris [23] claimed that the health impairment and the motivational process should be studied jointly. In that sense, workplace phobic anxiety could be included in the JD-R model, as it is an important descriptive concept for mental health at work, in addition to exhaustion [24]. In fact, as reported by Bakker et al. [22] there is evidence that job demands are the main antecedents of burnout, which in turn leads to poor health (such as workplace phobic anxiety) and negative organizational outcomes (such as absenteeism) and that job resources are the main causes of work engagement, which in turn leads to increased well-being (such as reduced workplace phobia) and positive organizational outcomes (such as decreased absenteeism).

Thus, the aim of this study is to analyse the concept of workplace phobic anxiety for the first time in a larger nonclinical sample (as recently suggested by Muschalla and Linden [6]) using jointly the health impairment and the motivational processes of the JD-R model.

Developing knowledge about workplace phobic anxiety in nonclinical samples will be important in order to raise awareness in organizations concerning this specific disease and subsequently to provide organizational strategies in order to prevent it.

In order to reach this aim, job demands and job resources have to be identified. The antecedents of burnout and work engagement (e.g., job demands and job resources) are described in two categories [22]: situational factors (e.g., workload) and individual factors (e.g., neuroticism, self-efficacy). As workplace phobic anxiety is context-specific, we argue that situational factors should be more appropriate for analyzing workplace phobic anxiety in organizational contexts. Furthermore, in order to choose the appropriate job demands and job resources, three main criteria have been followed: (a) selecting among the most used; (b) choosing the more non-occupation-specific ones; (c) identifying in the literature elements that could be more related to workplace phobic anxiety. Thus, according also to previous and established models on workers' well-being (e.g., Job Demand-Control model (JDC) [25]; later became the Job Demand-Control-Support model (JDCS) [26]) physical and psychological demands as job demands and social support from colleagues and from supervisors as perceived job resources are chosen. This is in line with the previous description of the context factors of work, which may interact with workplace phobic anxiety. In fact, workplace phobic anxiety may occur in comorbidity with specific social anxiety towards a specific superior or colleague [4]. Further, patients with workplace phobia reported more often being overtaxed at work because of the content or amount of work [6]. We included psychological demands as there is evidence that this dimension is related to anxiety disorders in both men and women [27]. Moreover, as one characteristic of the JD-R model is its flexibility, which means that demands and resources could be tailored to the specific occupation under consideration [20], we choose physical demands as the sample is composed by retail workers, which are exposed to biomechanical and ergonomic risk factors [28].

### 1.4. Research Question

In this present study we analyse the concept of workplace phobic anxiety using jointly the health impairment and the motivational processes of the JD-R model. We did this study with a sample of nonclinical Italian employees.

The following main hypotheses will be tested.

*Hypothesis 1.* The health impairment and motivational processes are significantly related to workplace phobic anxiety and subsequently to absenteeism. Particularly, we have the following.

*Hypothesis 1a.* Exhaustion mediates the relationship between perceived job demands (psychological and physical demands) and workplace phobic anxiety.

*Hypothesis 1b. *Work engagement mediates the relationship between perceived job resources (social support from colleagues and from supervisors) and workplace phobic anxiety.

*Hypothesis 1c. *Workplace phobic anxiety will be positively related to absenteeism.

## 2. Methods

### 2.1. Participants and Design

A cross-sectional survey was conducted in a large retail company in Italy. All the workers of the company were informed about the start of the project with an article published in the company journal. Then, 1000 workers randomly selected from the company database were invited to participate in the study by an e-mail sent by their supervisors. Workers were assembled in groups and asked to fill in a self-administered questionnaire. During the session, the researchers provided information about work-related stress and the project aims. Workers were invited to fill out the questionnaire but they were not obliged to do it. Furthermore, the researchers informed the participants that the employer would not be informed about the employees who decided not to take the survey. The questionnaire included a statement regarding the personal data treatment, in accordance with the Italian privacy law (Law Decree DL-196/2003). With regard to ethical standards for research, the study adhered to the latest version of the Declaration of Helsinki [29]. A researcher was always present during the session in order to clarify potential doubts concerning the questionnaire's items or the study in general. In total, 739 workers voluntarily participated in the study (the response rate was 73.9%). The majority of the participants (62.4%) were female and the mean age was 44.5 years (SD = 7.93). Organizational tenure mean was 17.03 years (SD = 8.79). Most of the participants (93%) had permanent contracts.

### 2.2. Measures

Consistent with the JD-R model, the questionnaire consisted of scales referring to job demands (physical and psychological demand), job resources (social support from supervisor and from colleagues), burnout, work engagement, and workplace phobia. Details about scales included in the questionnaire and absenteeism are presented below.

#### 2.2.1. Psychological Demand and Physical Demand

These were both measured with the Karasek's [30] Job Content Questionnaire. The scale consists of nine items with response options ranging from 1* (strongly disagree)* to 4* (strongly agree)*. One example of psychological demand is, “My job requires working very hard.” An example of physical demand is, “My job requires lots of physical effort.” Items were then averaged.

#### 2.2.2. Social Support from Supervisor/Colleagues

These dimensions were measured with the four-item scale of JCQ [30]. Answers ranged from 1* (strongly disagree)* to 4* (strongly agree)*. An example of social support from supervisor is, “My supervisor is concerned about the welfare of those under him”; an example of social support from colleagues is, “People I work with are helpful in getting the job done.” Items were then averaged.

#### 2.2.3. Exhaustion

It is measured as emotional exhaustion, which is the main component of burnout and refers to “feelings of being overextended and depleted of one's emotional and physical resources” ([31], p. 399). Emotional exhaustion was measured using the scale of the Maslach Burnout Inventory (MBI) ([13, 32]). The five items were scored on a 7-point frequency Likert scale (0 = “never” to 6 = “every day”) and then summed.

#### 2.2.4. Work Engagement

This is a multidimensional construct defined as a positive, fulfilling, work-related state of mind which is characterized by (a) vigor, that is, high level of energy and mental resilience while working, the willingness to invest effort in one's work, and persistence even in the face of difficulties (one example item is, “At my work, I feel bursting with energy”); (b) dedication, which refers to a sense of significance, enthusiasm, inspiration, pride, and challenge (one example item is, “I am proud of the work that I do”); (c) absorption, which is characterized by fully concentrating on and being deeply engrossed in one's work, where time passes quickly and one has difficulty detaching oneself from work ([33], p. 166). In this study the short version of the Utrecht Work Engagement Scale ([34]; Italian version by Balducci and colleagues [35]) was used, consisting of three items for each dimension previously described. All nine items were scored on a 7-point scale ranging from “0” (never) to “6” (always) and then averaged.

#### 2.2.5. Workplace Phobic Anxiety

In order to measure this construct, the Muschalla and Linden ([14, 36]) Workplace Phobia Scale was used. In this study, the Italian version was used [37] which consists of 12 items scored on a 5-point frequency Likert scale (1 = “do not agree at all” to 5 = “totally agree”). One example item is, “When imagining having to pass a complete working day at this workplace, I get feelings of panic.” The scale measures the degree of workplace phobic anxiety. In the following, we use the term “workplace phobic anxiety,” because no clinical diagnosis of workplace phobia has been made by a physician (implications about this will be presented in the discussion section). Items were averaged.

#### 2.2.6. Absenteeism

Based on the results of the Johns and Al Hajj [19] study, absenteeism was measured in terms of both duration (number of days a worker has been absent during one year) and frequency (number of absence spells during one year). A period of one year was chosen because it increases stability in the absence measures [38]. The organization provided the objective data of sickness leave duration and frequency for all of the participants in the study. The mean absence duration was 14.27 days (SD = 23.32; min = 0; MAX = 184) and most of the participants (75.2%) had been absent from work at least one day. The mean of absence frequency was 2.61 (SD = 3.27; min = 0; MAX = 22). Since both absence duration and absence frequency showed a considerable skewness (3.55 and 2.37, resp.) and kurtosis (16.20 and 6.95, resp.), a log⁡10 transformation was performed in order to approach a normal distribution [39].

### 2.3. Data Analysis

In order to test our hypothesis, structural equation modelling methods were employed using the AMOS software package version 21.0 with maximum likelihood estimation methods.

Exhaustion and workplace phobic anxiety were included as a single indicator (the average total score of the corresponding scale). In this case the error variance was estimated by using the formula (1 − *α*)*∗σ*^2^ [40]. The job demands variable was indicated by psychological demand and physical demand, while job resources variable was indicated by social support from colleagues and supervisors. Work engagement was indicated by vigor, dedication, and absorption and absenteeism was indicated by frequency and duration.

As job demands and job resources frequently correlate, meaning that high job resources could reduce job demands, and high job demands could prevent the mobilisation of job resources [21], job demands and job resources were related.

In order to test our hypotheses, several models were compared by means of Chi-squared differences tests [41]. As Chi-squared is sensitive to sample size, using relative goodness-of-fit measures is strongly recommended [42].

Thus, to establish the model's fit to the data, the following indexes were used: *χ*^2^ goodness of fit statistic; the Comparative Fit Index (CFI [42]); the Tucker-Lewis Index (TLI [43]); and the Root Mean Square Error of Approximation (RMSEA [44]). Fits can be considered acceptable when CFI and TLI are greater than 0.90 and the RMSEA is equal to or less than 0.08 [42, 45].

## 3. Results

In order to analyse the role of workplace phobic anxiety as a health outcome in the JD-R model, preliminary and structural equation model analyses were performed.

### 3.1. Preliminary Analyses

Means, standard deviations, reliabilities, and Pearson correlations are shown in Table 1. All the scales used showed a good reliability and satisfied the criterion of .70 [46] except for scales concerning the absorption dimension of work engagement (.63) and social support from colleagues (.67). Comparing our results on workplace phobic anxiety with previous studies [20], most people in this present study had no clinically relevant work-anxiety (84.8%, M: 1–2.5), some had moderate work-anxiety (11.1%, M: 2.51–3.5), and 4.1% had severe work-anxiety (M > 3.5). This is similar to the rate of 5% who report avoidance due to work-anxiety in the German study [20].

All the correlation results were in the expected direction and all the values showed a significant association except for the relationship between psychological demand and dedication, absorption, and sickness duration. Note that workplace phobic anxiety is only partially related to emotional exhaustion (*r* = .54), which is the central component of burnout. This result suggests that workplace phobic anxiety is different from burnout.

### 3.2. Structural Equation Modelling

In order to test our hypotheses, structural equation modelling was computed. As shown in the first row of Table 2, the proposed model (M1) fits reasonably the data with all indexes meeting their respective criteria.

**Figure 1:**
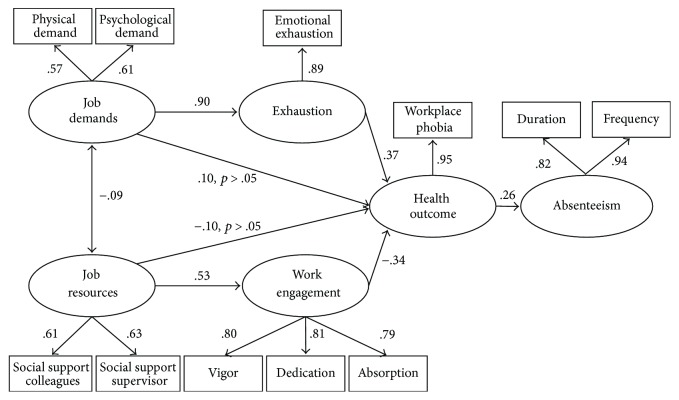
Standardized coefficients of the mediation model.

All structural paths between latent factors were significant and in the expected direction. In the next series of analyses, the full mediation model was compared with the partial mediation model, including direct paths from job demands and workplace phobic anxiety and from job resources and workplace phobic anxiety (M2). The results showed that the inclusion of these additional paths did not improve the model fit (Δ*χ*^2^(2) = 4.15,   *p* > .05). Consistent with this result, the paths from job demands to workplace phobic anxiety (*γ* = .10, *p* > .05) and from job resources to workplace phobic anxiety (*γ* = −.10, *p* > .05) were nonsignificant. All structural paths are depicted in Figure 1. Hypothesis 1c, workplace phobic anxiety is related to absenteeism, is confirmed.

Subsequent Sobel tests supported the mediating role of exhaustion in the relationship between job demands and workplace phobic anxiety (*z* = 2.11; *p* < .05) and supported the mediating role of work engagement in the relationship between job resources and workplace phobic anxiety (*z* = − 5.67; *p* < .001). Thus, all of the hypotheses have been confirmed.

## 4. Discussion

Results of this study suggested that workplace phobic anxiety (or workplace phobia, when diagnosed) can be considered as a health parameter of the JD-R model. Furthermore, in line with the two processes of the model (health impairment and motivation), exhaustion mediates the relationship between job demands and workplace phobic anxiety, and work engagement mediates the relationship between job resources and workplace phobic anxiety. Moreover, the results showed that workplace phobic anxiety is related to absenteeism, taking into account both duration and frequency.

According to the JD-R model, other studies suggested a relationship between exhaustion and health outcomes such as depressive symptoms and life satisfaction [47] and mood disturbance [48]. Furthermore, other recent studies investigated the mediating role of exhaustion in the health impairment process, considering job demands as predictors and outcomes such as absenteeism [49].

Nevertheless, to the best of our knowledge, this is the first study which considered a health related outcome specific for work, such as workplace phobic anxiety.

Findings of this study confirmed what was suggested by Schaufeli and Taris [23] that the health impairment process and the motivational process should be studied jointly. Particularly, results showed how perceived job demands are related to increased emotional exhaustion in the workers, which in turn is moderately associated with workplace phobic anxiety. Concerning the motivational process of the JD-R model, results provided evidences for the fact that perceived job resources are relevant for work engagement, which in turn is related to decreased workplace phobic anxiety.

Moreover, results of this study suggested that work engagement is associated with lower workplace phobic anxiety. Despite the relationship between job resources and health outcomes not being one of the most studied in the JD-R model, this result is in line with the findings of a study by Bakken and Torp [50]. In their study, a significant relationship between work engagement and health was found. Also, Schaufeli et al. [51] in their study of telecom managers found a negative association between two dimensions of work engagement (vigor and dedication) and anxiety, depression, and psychosomatic complaints.

Results presented in this study also confirm the relationship between workplace phobic anxiety and absence duration from work, meaning that the higher a workplace phobic anxiety, the higher the absenteeism (measured as both frequency and duration). The relationship between workplace phobic anxiety and absenteeism could be twofold [4]. On one side, anxiety can be manifested initially at the workplace, and sick leave occurs as a result of this. On the other side, the longer the duration of sick leave due to any (not strictly work-related) health issue, the more increased the perception of workplace-related anxiety. In other words, workplace phobic anxiety could develop as a result of extended sick leave, because of rising perceptions of uncertainty, or speculative anticipation of possible changes happening at work while the person is absent.

### 4.1. Strengths and Limitations

One of the main strengths of this study is using the underrecognized construct of workplace phobic anxiety. This is the first study that investigates workplace phobic anxiety and its association with other constructs in an organizational setting. Both subjective data (collected through questionnaires) and objective data (collected through the company's records on absence duration) were used for testing the hypotheses.

A limitation of the study is the cross-sectional design, which precludes causal relationships between the variables examined. Furthermore, the study was conducted in one specific organization; thus further evidence in other professional groups is needed for comparative purposes. The Workplace Phobia Scale was to measure the degree of workplace phobic anxiety as a dimensional construct. In future research, there should also be observer-ratings in the sense of a clinical diagnosis of workplace phobia done by interview [9] in order to find out the prevalence of workplace phobia in general and in specific contexts. Lastly, as workplace phobic anxiety is associated with absenteeism and data have been collected in group sessions at the workplace, it is plausible that some workers really affected by workplace phobic anxiety were absent during the data collection. Future studies could be conducted with online surveys, so that all the workers of the organization could have the chance to answer and to decrease potential bias in the sample.

### 4.2. Direction for Future Research, Practical Implications, and Conclusion

Future studies should be conducted using a longitudinal design and investigating various occupational settings. Longitudinal studies could also measure how workplace phobic anxiety could develop under various work environment factors or work environment changes. There is also a need for future studies that analyse the most important job demands, which could increase workplace phobic anxiety. For example, future studies could consider the potential occurrence of traumatic events in the workplace and the kind of jobs that could be more exposed to dangers (e.g., bank employees, workers in emergency rooms, or social services).

As the JD-R model posits that perceived job resources could buffer the impact of job demands on exhaustion, future studies should investigate also the kind of job resources that could decrease the impact on emotional exhaustion and, in turn, workplace phobic anxiety. Considering also the role played by personal resources in the JD-R model (e.g., [52]), future studies should investigate the personal resources that could play a role in determining workplace phobic anxiety. Moreover, future studies should investigate the existence of a potential reciprocal effect between perceived job demands, workplace phobic anxiety, absenteeism from work, and the potential worries of returning to work.

Results of this study suggested that workplace phobic anxiety is an important issue in organizations for both the workers' health and the organizational costs linked to absenteeism. Thus, organizations should be aware of this phenomenon. In line with Muschalla and Linden's [6] suggestion that primary care physicians should be aware of workplace phobia, also occupational physicians should be aware that it could be a case of workplace phobic anxiety when workers complain about bad work conditions [53] or are often (or for long periods) on sick leave. According to the JD-R model [22], results of this study suggest that organizations should implement interventions in order to optimise job demands and increase job resources such as support from the supervisor and colleagues, which are related to higher levels of work engagement and the development of workplace phobic anxiety symptoms. Furthermore, organizations could monitor the sickness leaves of their employees and implement interventions such as those mindfulness based, as a recent systematic review developed by Lomas and colleagues [54] showed how these interventions are able to reduce anxiety in workers.

## 5. Conclusion

This study focused on workplace phobic anxiety, which is a costly and disabling phenomenon in the occupational health context that needs further research [4]. Results suggest that workplace phobic anxiety is a specific concept and an important issue in organizations for both the health of workers and the organizational costs linked to absenteeism. Supervisors and occupational physicians should be aware of workplace phobic anxiety, especially when workers are often (or for long periods) on sick leave.

This study contributes to knowledge on workers' health in organizations and how psychosocial risk factors in the organizations could contribute to the development of anxiety disorders, such as workplace phobia.

## Figures and Tables

**Table 1:** Means, standard deviations, reliabilities, and Pearson correlations for all variables (*N* = 739).

Variables	M	SD	*α*	1	2	3	4	5	6	7	8	9	10
(1) Psychological dem.	2.80	.48	.70	—									
(2) Physical dem.	2.56	.81	.81	.36^*∗∗∗*^	—								
(3) Soc. Supp. Superv.	2.86	.77	.81	−.26^*∗∗∗*^	−.15^*∗∗∗*^	—							
(4) Soc. Supp. Coll.	2.96	.57	.67	−.23^*∗∗∗*^	−.19^*∗∗∗*^	.41^*∗∗∗*^	—						
(5) Emot. exhaustion	16.98	7.32	.79	.48^*∗∗∗*^	.45^*∗∗∗*^	−.28^*∗∗∗*^	−.25^*∗∗∗*^	—					
(6) Vigor	4.70	1.27	.77	−.12^*∗∗∗*^	−.17^*∗∗∗*^	.27^*∗∗∗*^	.23^*∗∗∗*^	−.36^*∗∗∗*^	—				
(7) Dedication	4.78	1.53	.87	−.07	−.18^*∗∗∗*^	.31^*∗∗*^	.25^*∗∗∗*^	−.29^*∗∗∗*^	.63^*∗∗∗*^	—			
(8) Absorption	4.99	1.09	.63	−.07	−.13^*∗∗∗*^	.21^*∗∗∗*^	.19^*∗∗∗*^	−.22^*∗∗∗*^	.64^*∗∗∗*^	.65^*∗∗∗*^	—		
(9) WPP	1.73	.79	.90	.31^*∗∗∗*^	.34^*∗∗*^	−.28^*∗∗∗*^	−.32^*∗∗∗*^	.54^*∗∗∗*^	−.47^*∗∗∗*^	−.44^*∗∗∗*^	−.35^*∗∗∗*^	—	
(10) Duration	.77	.60	—	.07	.16^*∗∗∗*^	−.14^*∗∗∗*^	−.12^*∗∗∗*^	.16^*∗∗∗*^	−.17^*∗∗∗*^	−.17^*∗∗∗*^	−.14^*∗∗∗*^	.20^*∗∗∗*^	—
(11) Frequency	.29	.34	—	.09^*∗*^	.15^*∗∗∗*^	−.19^*∗∗∗*^	−.11^*∗∗*^	.19^*∗∗∗*^	−.21^*∗∗∗*^	−.23^*∗∗∗*^	−.16^*∗∗∗*^	.22^*∗∗∗*^	.77^*∗∗∗*^

*Notes.* ^*∗*^ *p* < .05; ^*∗∗*^*p* < .01; ^*∗∗∗*^*p* < .001.

**Table 2:** Fit of model composed by workplace phobic anxiety as a health outcome of the JD-R model (*N* = 739).

Model	*χ* ^2^	df	TLI	CFI	RMSEA	Model comparison	Δ*χ*^2^	Δdf
M1. hypothesized model	141.66^*∗∗∗*^	40	.95	.96	.06	—	—	—
M2. partial mediation model	137.51^*∗∗∗*^	38	.95	.96	.06	M1-M2	4.15	2

*Notes. χ* ^2^ = Chi-squared, df = degrees of freedom; TLI = Tucker-Lewis Index; CFI = Comparative Fit Index; RMSEA = Root Mean Square Error of Approximation; ^*∗∗∗*^*p* < .001.
